# Editorial: Glial cells in homeostasis, neurodevelopment, and repair

**DOI:** 10.3389/fncel.2025.1575105

**Published:** 2025-02-27

**Authors:** Antonio Cibelli, David C. Spray, Maria Grazia Mola

**Affiliations:** ^1^Department of Biosciences, Biotechnology and Environment, University of Bari Aldo Moro, Bari, Italy; ^2^Dominique P. Purpura Department of Neuroscience, Albert Einstein College of Medicine, New York, NY, United States; ^3^Department of Medicine and Surgery, Libera Università Mediterranea ”Giuseppe Degennaro“, Casamassima, Italy

**Keywords:** glial cells, neurodevelopment, CNS homeostasis, water channels, ion channels, biomaterial

The widely accepted concept of the neurovascular unit suggests that proper brain function relies on dynamic communication and homeostatic signaling among neurons, glial cells, and vascular compartment. This Research Topic aims to deepen the understanding of the molecular mechanisms and functional dynamics of these cells, shedding light on brain homeostasis alterations caused by ischemic or traumatic injuries and neurodegenerative diseases. Additionally, it seeks to give new insights into the development of effective therapeutic strategies.

Glial cells, including astrocytes, microglia and oligodendrocytes, play crucial roles in regulating synaptic function, providing metabolic support, forming myelin sheaths for signal transduction, and contributing to the immune response of the central nervous system (CNS) (Liu et al., 2023). Additionally, they are key players in disease and post-traumatic recovery processes: microglia serve as the primary source of pro-inflammatory cytokines (Conti et al., 2020), astrocytes regulate synaptic homeostasis, permeability of the blood-brain-barrier to water and solutes and glial scar formation following injury (Chung et al., 2024; Sofroniew, 2009; Pekny et al., 2019), while oligodendrocytes influence signal conduction speed through changes in myelin thickness (Miyata, 2019).

The systematic review by Amlerova et al. summarizes the current knowledge on the complex molecular and cellular processes of reactive gliosis following traumatic brain injury (TBI), highlighting its dual role of providing neuroprotection but also being potentially harmful due to the release of growth factors and cytokines by reactive glia. Indeed, while reactive gliosis aids tissue repair, immune modulation and homeostasis, it can also exacerbate neuroinflammation and neurological deficits. Given that current therapies mainly address symptoms rather than targeting the molecular and cellular mechanisms of damage, the ability to modulate neuroinflammatory processes would be crucial to develop effective therapeutic approaches that balance their beneficial or detrimental effects. Glia-targeted treatments are currently promising, such as senolytic compounds that target senescent cells to improve cognition, and transcription factors used to reprogram astrocytes or NG2 glia and support post-injury neurogenesis.

Microglia are the major population of immune cells resident in the CNS and play a fundamental role in the response to injury, regulating neuroinflammation, clearing cellular debris and thereby providing neuroprotection (Kettenmann et al., 2011). Dysregulation of microglial activity is associated with several neurodevelopmental, neurological and psychiatric disorders (Hickman et al., 2018). The review by Bobotis et al. provides a comprehensive overview of established and emerging technologies to characterize the complex morphological and functional heterogeneity of microglia in homeostasis and disease. Innovative technologies, including single-cell transcriptomic and epigenomic analyses, have revealed that both astrocytes and microglia comprise heterogeneous cellular subpopulations with distinct genomic and functional characteristics that vary according to the physiological and pathological context and play major roles in neurodegeneration and neuroprotection. Depending on the representation of specific glial subpopulations, tissue damage, regenerative processes, or delayed neurodegeneration after TBI may vary between nearby or remote areas and between different brain structures. Parallel to advances in microglial biology, the reviews by Bobotis et al. and by Amlerova et al. offer a broad discussion of emerging techniques of depletion and selective modulation of microglial activity as potential therapeutic approaches for neurodegenerative, neuropsychiatric and autoimmune diseases.

Since neuronal regeneration is mostly limited following CNS injuries and diseases, glial cells have emerged as crucial targets for the development of active pharmaceutical ingredients (APIs) aimed at improving clinical outcomes (Madadi and Sohn, 2024). Although many of these APIs demonstrate potential *in vitro*, when systemically delivered most APIs exhibit limited penetration through the blood-brain barrier (BBB) or blood-spinal cord barrier (BSCB), making them ineffective. To address this Research Topic, nanomaterials have been engineered to transport APIs to their target sites, extend the release timeframe, and influence cellular behavior through their structural properties (Ciciriello et al., 2022). The review by Saksena et al. explores the latest advancements of both locally implanted nanomaterials and systemically administered nanoparticles developed for the delivery of APIs to CNS glia, highlights existing research gaps, and discusses future developments that could sustain this approach toward clinical applications. As many API-releasing nanomaterials remain focused on targeting neurons, further advancements in material engineering, BBB permeability, targeted delivery, controlled release, and immune response management are essential to establish nanomaterial-mediated API delivery to glia as a standard clinical approach. This review summarizes emerging strategies for delivering APIs to astrocytes, microglia, and oligodendrocytes using nanomaterials. Nanoparticle-based therapies present a non-invasive alternative for neurological conditions where direct implantation is not feasible, with optimization of nanoparticle composition, size, charge, and targeting mechanisms being crucial for effective brain penetration and glial modulation. In surgical interventions, nanomaterial-based therapies provide biophysical and biochemical cues to glial cells, stimulating bioactive responses that enhance neuronal repair and protection.

Optogenetics is a cutting-edge technique that combines genetic engineering with light-based stimulation to fine-tune neuronal activity (Towne and Thompson, 2016). This method relies on incorporating light-sensitive proteins, called opsins, into specific neurons or brain regions, enabling precise activation or inhibition through photostimulation. Through optogenetic manipulation, researchers can precisely control neuronal activity in both space and time, offering critical insights into neural circuits and brain function (Fernandez-Ruiz et al., 2022). Moreover, optogenetic treatment enhances neuronal excitability and synaptic responses, contributing to the maintenance of nervous system health. As a result, optogenetics holds significant potential for applications in neuroscience research. Hyung et al. reviews the optogenetic manipulation of different glial cell populations and its impact on the reciprocal neuro-glial interaction in neural repair. Since damaged neurons do not regenerate independently and rely on glial cells for structural and trophic support, the application of glial optogenetics appears to be a promising approach for nerve repair. Current research aims to determine whether modulation of glial cells via optogenetics can more effectively regulate neural function than direct neuronal activation, as well as to identify the optimal level of opsin gene expression required to stimulate glial cells and sustain the excitatory activity of neurons. However, several challenges remain, including assessing whether glial optogenetic modulation can correct nervous system dysfunction and assessing whether the effects of optogenetic activation are consistent *in vitro, in vivo*, and ultimately in clinical settings.

Advancements in stroke research, combined with progress in imaging technology, have paved the way for new approaches in managing acute ischemic stroke patients. Researchers in the field of stroke investigation are striving to uncover strategies that can prevent episodes of focal ischemia, enhance the quality of life for survivors, and mitigate premature mortality (Saceleanu et al., 2023; Herpich and Rincon, 2020). To succeed in this effort, it is imperative to broaden the perspective beyond the confines of primary injury sites and adopt a holistic approach, viewing the brain as an interconnected system where all components influence each other. Therefore, as highlighted by Koukalova et al. it is crucial to focus both on the ischemic core and on the adjacent, initially undamaged regions, which are susceptible to the development of secondary lesions that can manifest subtly and over time. In their literature review, Koukalova et al. provide an exhaustive description of the terms “ischemic core”, “penumbra” and “remote areas” specifically emphasizing the structural and functional changes that occur in regions distant from the primary site of focal ischemia and on the involvement of glia and the extracellular matrix. The processes that commonly affect the ischemic core and the penumbra are compared and the causes analyzed. Special attention is paid to the investigative approaches and the efficacy of therapies used in recent years for their anti-ischemic effect in remote areas of the lesion. Studying glial responses in these areas could lead to a better understanding of the mechanisms underlying secondary neurodegeneration and new strategies to counteract it.

Astrocytic Transient Receptor Potential Vanilloid 4 (TRPV4) channels, along with Aquaporin 4 (AQP4), are believed to play a crucial role in cellular volume regulation (Pivonkova et al., 2018; Mola et al., 2021; Barile et al., 2023; Cibelli et al., 2024) and may influence the onset and severity of cerebral edema during ischemia (Chmelova et al., 2019; Sucha et al., 2022). The original research article by Hermanova et al. investigates the impact of AQP4 and TRPV4 channel deletion on astrocytic volume changes in response to three ischemia-mimicking insults. Their findings revealed that cortical astrocytes exhibited heterogeneous volume responses to pathological stimuli, with a high-responding astrocyte (HRA) subpopulation being affected more by the loss of AQP4, TRPV4, or both channels. While AQP4 deletion reduced swelling during oxygen-glucose deprivation (OGD), TRPV4 deletion delayed its onset. Surprisingly, simultaneous deletion had minimal impact on volume regulation but impaired recovery after OGD. Additionally, knockout of these channels altered the expression of glutamate receptors and ion channels, suggesting broader molecular effects on cerebral edema beyond their direct role in volume regulation.

This Research Topic compiles new knowledge regarding the strategic role of glial cells in brain homeostasis, development, and recovery from neural injury while integrating optogenetic, physiological, molecular, and engineering investigative approaches to provide new insights into multiple areas of cellular neuroscience. Despite significant advances, many fundamental neuroscientific challenges remain unsolved. We hope that this Research Topic will support researchers in challenging existing paradigms, expanding the frontiers of neuroscience, and ultimately contributing to the development of novel and effective therapies in neurology.
